# Acute effects of different percussive massage frequencies on shoulder proprioceptive recovery and upper-limb performance in male artistic gymnasts: a randomized crossover trial

**DOI:** 10.3389/fphys.2026.1996859

**Published:** 2026-10-28

**Authors:** Hao Wang, Sihan Jiao, Maiwulanjiang Tuerhongjiang, Fangyu Zhao, Bin Shao

**Affiliations:** 1 School of Education, Beijing Sport University, Beijing, China; 2 Sports Coaching College, Beijing Sport University, Beijing, China; 3 Physical Education School, Shanxi Normal University, Xian, China; 4 School of Physical Education, Shanghai University of Sport, Shanghai, China

**Keywords:** artistic gymnastics, joint position sense, percussive massage, proprioception, range of motion, recovery

## Abstract

**Purpose:**

This study compared the acute effects of different percussive massage (PM) frequencies on post-fatigue shoulder proprioceptive recovery, perceived fatigue, range of motion (ROM), and upper-limb explosive performance in high-level male artistic gymnasts.

**Methods:**

Fifteen gymnasts completed three recovery conditions in a randomized crossover design: 10 min of seated rest (CTRL), 30 Hz PM, and 60 Hz PM, with a 1-week washout period between sessions. Upper-limb fatigue was induced using an individualized handstand push-up protocol. Shoulder internal- and external-rotation joint position sense (JPS), shoulder flexion ROM, and ballistic push-up performance were assessed at baseline (P0), after fatigue induction (P1), and after recovery (P2); subjective fatigue was assessed at P1 and P2.

**Results:**

Both PM conditions produced greater reductions in subjective fatigue than CTRL (both p < 0.001), with a greater reduction following 60 Hz than 30 Hz PM (p = 0.038). At P2, internal- and external-rotation JPS errors were lower after both PM conditions than after CTRL (p ≤ 0.008), and were lower after 30 Hz than 60 Hz PM (p ≤ 0.016). Shoulder flexion ROM increased from P1 to P2 following both PM conditions (p ≤ 0.001), but no significant between-condition differences were observed. No condition-specific effects were found for peak force, peak power, or concentric-phase impulse.

**Conclusion:**

PM produced outcome- and frequency-dependent recovery responses following upper-limb fatigue. The 60 Hz condition was more effective for reducing perceived fatigue, whereas 30 Hz PM produced greater recovery of shoulder proprioception. Neither frequency provided an additional benefit for upper-limb explosive performance.

## Introduction

Artistic gymnastics (AG) requires strength, balance, precise execution, rapid force production, and effective whole-body control (Kara, 2021). In men’s AG, athletes compete across multiple apparatuses and repeatedly perform high-intensity upper-limb support, swinging, pushing, and landing-related actions within relatively short recovery intervals (Kaufmann et al., 2022). The shoulder joint is extensively involved in these tasks and plays a central role in maintaining sensorimotor control during support, swing, and transition movements. In particular, accurate shoulder proprioception is essential for detecting joint position and regulating movement, whereas sufficient shoulder range of motion (ROM) and upper-limb force-generating capacity support the execution of large-amplitude and explosive gymnastics skills (Milosis, 2023; Yu et al., 2024).

Repeated high-intensity routines during training and competition may induce substantial upper-limb fatigue and disturb the sensorimotor processes required for precise shoulder control. Exercise-induced fatigue can reduce proprioceptive acuity, alter proprioceptive afferent input, and impair neuromuscular control, which may compromise the ability to accurately reproduce joint positions and stabilize the shoulder during subsequent movements (Proske, 2019). Fatigue may also reduce joint mobility and force-generating capacity, further limiting the execution of technically demanding skills (Isacco et al., 2019). These effects are particularly relevant in artistic gymnastics, where athletes may have only limited time to recover between apparatus rotations (Jemni, 2018). Accordingly, recovery strategies that can rapidly restore both sensorimotor function and physical performance may be especially valuable in this setting.

Percussive massage (PM) has emerged as a practical recovery modality that delivers repetitive mechanical stimulation to targeted muscles through a handheld device. Beyond its potential effects on perceived discomfort and joint mobility, the mechanical stimulation generated by PM may also influence peripheral sensory input, tissue mechanical properties, and neuromuscular function. Repetitive percussion may stimulate cutaneous, myofascial, and muscle-related mechanoreceptors, while transient changes in muscle stiffness and tissue compliance may modify the mechanical environment in which proprioceptive signals are generated (Cheatham et al., 2021). Previous studies have reported acute increases in ROM and changes in perceived discomfort, muscle mechanical properties, and neuromuscular responses following PM (Konrad et al., 2020; Ferreira et al., 2023; Skinner et al., 2023). These findings provide a physiological rationale for considering PM as a potential strategy for post-fatigue sensorimotor recovery. However, its effects on proprioceptive recovery following sport-specific upper-limb fatigue remain poorly understood.

An additional issue is whether the physiological and functional responses to PM depend on the characteristics of the mechanical stimulus. Percussion frequency determines the temporal pattern of mechanical stimulation delivered to the treated tissues and may therefore differentially influence sensory afferent input, tissue mechanical responses, and perceived recovery. Mechanical vibration can interact with the mechanical and neuromuscular properties of soft tissues, and responses may vary according to stimulation frequency (Wakeling et al., 2002; Yuan, 2022). Nevertheless, the frequency dependence of PM-induced recovery remains unclear. Most previous studies have focused on lower-limb muscles or generally active populations, whereas little is known about whether different PM frequencies produce distinct recovery responses in the fatigued shoulder of highly trained gymnasts. This question is particularly relevant because recovery of proprioceptive function, joint mobility, perceived fatigue, and explosive performance may rely on partly distinct physiological processes and therefore may not respond uniformly to the same mechanical stimulus.

Therefore, this randomized crossover study aimed to compare the acute effects of different PM frequencies on perceived fatigue, shoulder joint position sense, shoulder ROM, and upper-limb explosive performance following exercise-induced fatigue in high-level male artistic gymnasts. We hypothesized that PM would facilitate post-fatigue recovery compared with passive rest, but that the magnitude of recovery would differ across outcomes and according to PM frequency.

## Methods

The study protocol was approved by the Sports Science Experiment Ethics Committee of Beijing Sport University (approval No. 2025178H). All participants provided written informed consent before participation. The study was conducted in accordance with the Declaration of Helsinki.

### Participants

An *a priori* sample size calculation was performed using G*Power 3.1 based on effect sizes reported in previous related studies (Custer et al., 2017). The calculation was conducted for a repeated-measures analysis of variance (ANOVA) with within-subject factors, assuming an effect size of f = 0.30, a significance level of α = 0.05, and statistical power of 0.80. The required sample size was 12 participants. Allowing for an anticipated dropout rate of approximately 20%, 15 high-level male artistic gymnasts were recruited (age: 21.27 ± 2.15 years; height: 171.16 ± 4.38 cm; body mass: 65.32 ± 6.43 kg; artistic gymnastics training experience: 10.67 ± 4.82 years; handstand push-up repetition maximum: 22.07 ± 3.35).

Participants were eligible if they (i) were classified as Tier 2 (Highly Trained/National Level) (McKay et al., 2022); (ii) were free from neuromuscular injuries; (iii) had not regularly used a percussive massage device for warm-up or recovery before the study; and (iv) had maintained their usual lifestyle during the month preceding the experiment and refrained from high-intensity training for at least 48 h before each experimental session. Throughout the study, participants recorded their dietary intake and daily activities and were instructed to avoid alcohol, caffeine, and nutritional supplements that might influence the experimental outcomes. Participant flow is illustrated in **Figure 1**.

**Figure 1:**
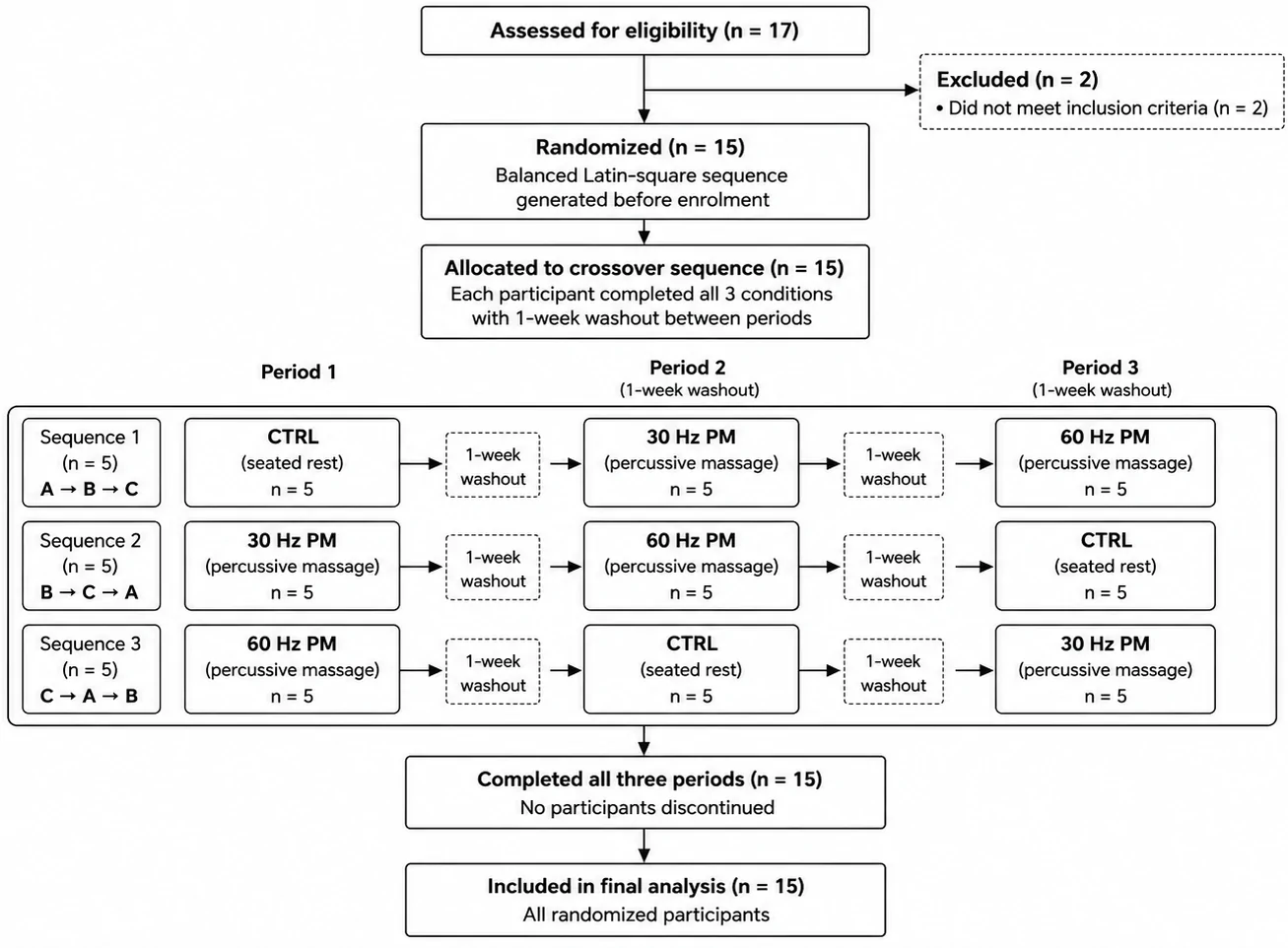
Flow diagram of participant recruitment, randomization, crossover allocation, and analysis. Seventeen male artistic gymnasts were assessed for eligibility, of whom two were excluded for not meeting the inclusion criteria. The remaining 15 participants were allocated to three crossover sequences according to a balanced Latin-square design and completed all three experimental conditions, with a 1-week washout period between consecutive sessions. All participants completed the study and were included in the final analysis. CTRL, seated-rest control; PM, percussive massage; A, CTRL; B, 30 Hz PM; C, 60 Hz PM.

### Study design

A randomized crossover controlled trial was conducted. Following a familiarization session, participants completed three experimental conditions: (i) 10 min of seated rest (CTRL), (ii) 30 Hz percussive massage (30 Hz), and (iii) 60 Hz percussive massage (60 Hz). The order of the three conditions was assigned using a balanced Latin-square design to minimize potential order effects. Because of the nature of the interventions, participants and the therapist delivering the percussive massage were aware of the recovery condition. A one-week washout period separated consecutive experimental sessions to reduce potential carryover effects. To minimize potential circadian influences, each participant completed all experimental sessions at the same time of day.

On each experimental day, participants rested quietly in a seated position for 5 min upon arrival before completing a standardized warm-up. Baseline measurements (P0) were then obtained, with a 1-min seated rest between outcome assessments. Participants subsequently completed the individualized fatigue protocol. Immediately afterward, subjective fatigue was assessed using the VAS, followed by shoulder joint position (JPS) sense (2 min), shoulder flexion ROM (1 min), and ballistic push-up performance (2 min), with 1-min rest intervals between the three tests (P1). Participants then underwent the allocated recovery condition for 10 min. The intervention duration was selected to reflect the typical recovery interval between apparatus rotations during men’s artistic gymnastics competitions. Immediately after the 10-min recovery period, VAS was assessed, followed by the same testing sequence and rest intervals (P2). The sequence of assessments, test durations, and inter-test rest intervals during each experimental session are illustrated in **Figure 2**.

**Figure 2:**
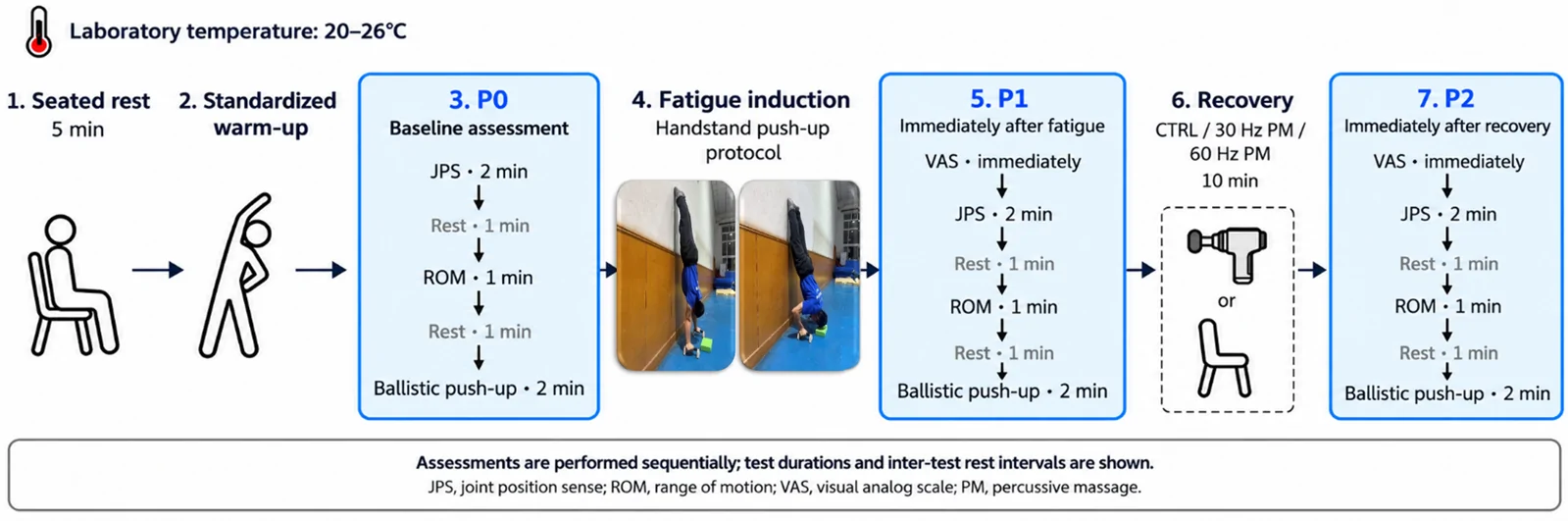
Experimental session timeline and assessment sequence.

### Percussive massage intervention protocol

Percussive massage (PM) was administered using a handheld percussive massage device (Hypervolt 2.0, Hyperice, USA) equipped with a spherical attachment. The device was operated at either 30 Hz or 60 Hz with a fixed amplitude of 2 mm and a sinusoidal vibration waveform (Masud et al., 2022). Participants were positioned either seated or supine, depending on the muscle group being treated.

To minimize inter-operator variability, all PM interventions were delivered by the same licensed rehabilitation therapist. The therapist was not involved in outcome assessment. During treatment, the massage head was maintained perpendicular to the muscle surface and moved longitudinally along the direction of the muscle fibers. No additional external pressure was applied beyond that required to maintain continuous contact between the massage head and the skin. The target muscle groups and treatment durations are presented in **Table 1**.

**Table 1:** Target muscle groups, anatomical attachments, intervention duration, and body position.

Target muscle group	Anatomical attachments	Intervention duration	Body position
Deltoid	Origin: lateral third of the clavicle, acromion, and spine of the scapula; insertion: deltoid tuberosity of the humerus	60 s/side	Seated
Long head of biceps brachii	Origin: supraglenoid tubercle of the scapula; insertion: radial tuberosity	30 s/side	Supine
Long head of triceps brachii	Origin: infraglenoid tubercle of the scapula; insertion: olecranon of the ulna	30 s/side	Seated, with the elbow flexed at 90°
Brachioradialis	Origin: lateral supracondylar ridge of the humerus and lateral intermuscular septum of the arm; insertion: distal radius, proximal to the styloid process	30 s/side	Seated, with the elbow flexed at 90° and the shoulder abducted at 45°
Pectoralis major	Origin: medial half of the clavicle, sternum, upper costal cartilages, and aponeurosis of the external oblique; insertion: lateral lip of the intertubercular sulcus of the humerus	120 s	Supine
Latissimus dorsi	Origin: spinous processes of T7–T12, thoracolumbar fascia, iliac crest, and lower ribs; insertion: floor of the intertubercular sulcus of the humerus	120 s	Seated

### Exercise-induced fatigue protocol

A standardized handstand push-up protocol was adopted to induce upper-limb exercise-induced fatigue and to replicate the sport-specific demands of male artistic gymnasts (van Dieën et al., 2012; Pau et al., 2021).

During the familiarization session, each participant’s handstand push-up repetition maximum was determined and subsequently used to individualize the exercise-induced fatigue protocol. Participants performed the exercise facing a wall in an inverted position, with both feet maintained in contact with the wall throughout the test. The handstand blocks were positioned 25–30 cm from the wall, and the hands were placed slightly wider than shoulder width. Movements were paced by a metronome at 15 repetitions·min^-1^, with each repetition consisting of a 3-s eccentric phase followed by a 1-s concentric phase. During the eccentric phase, the forehead was required to touch a preset marker, whereas full elbow extension was required during the concentric phase.

The repetition maximum (RM) handstand push-up test was terminated when participants either (i) failed to satisfy the movement criteria for two consecutive repetitions or maintain the prescribed cadence of 15 repetitions·min^-1^, or (ii) voluntarily indicated that they were unable to continue. The resulting RM value was subsequently used to individualize the fatigue protocol.

During each experimental session, the target number of handstand push-up repetitions per set was calculated as 60% of each participant’s individual repetition maximum, rounded down to the nearest whole number (e.g., 12 × 0.60 = 7.2, yielding 7 repetitions per set). Sets were separated by 1 min of rest and repeated until a predefined fatigue criterion was met. Exercise-induced fatigue was considered to have occurred when one of the following criteria was met: (i) heart rate exceeded 85% of the age-predicted maximal heart rate, calculated as 207 – 0.7 × age (Gellish et al., 2007); (ii) rating of perceived exertion reached ≥ 17 (“very hard”) (Wang, 2025); or (iii) the participant failed to satisfy the movement criteria for two consecutive repetitions or maintain the required frequency of 15 repetitions·min^-1^ (Xue, 2023).

### Shoulder internal and external rotation joint position sense

Shoulder internal- and external-rotation JPS of the dominant limb was assessed using an active joint position reproduction protocol on a multi-joint isokinetic dynamometer (ISOMED 2000), following procedures adapted from previous studies (Hong et al., 2010). Participants were seated with the dominant shoulder abducted to 90° and the elbow flexed to 90°, which served as the starting position (0°). Target positions were set at 45° of shoulder internal rotation and 45° of shoulder external rotation.

Before testing, the dynamometer was calibrated according to the manufacturer’s instructions. Participants wore an eye mask and earplugs to eliminate visual and auditory feedback. During familiarization, participants actively moved from the starting position to each target angle and maintained the position for 5 s to perceive and memorize the joint position.

During testing, participants actively reproduced each target angle at a comfortable, self-selected speed. To minimize the possibility of estimating the target position from movement duration, the dynamometer velocity limit was randomly set between 40 and 60°·s^-1^. Participants were instructed to stop the movement when they perceived that the target position had been reached, after which the reproduced angle was automatically recorded by the dynamometer. Three trials were completed for both internal and external rotation, with 5 s of rest between trials. JPS error was calculated as the absolute difference between the reproduced and target angle, and the mean absolute error across the three trials was used for statistical analysis.

### Shoulder flexion range of motion

Shoulder flexion ROM was assessed using the prone shoulder flexion test according to the physical fitness assessment guidelines for male artistic gymnasts (Sleeper et al., 2016). Participants lay in a prone position with the chin and tip of the nose in contact with the floor. Both arms were fully extended overhead while holding a gymnastics stick with the hands positioned shoulder width. Participants were instructed to actively raise both arms to the highest possible position while maintaining full elbow extension and continuous contact between the chin, nose, and floor.

The maximal position was briefly maintained to allow measurement. The vertical distance from the lower edge of the gymnastics stick to the floor was measured to the nearest 0.5 cm and used to represent shoulder flexion ROM. Three trials were completed with 10 s of rest between trials, and the mean value of the three trials was used for statistical analysis.

### Upper-limb explosive performance

Upper-limb explosive performance was assessed using two force platforms (Kistler 9287C, Switzerland) synchronized with BioWare software at a sampling frequency of 1000 Hz, following a previously described ballistic push-up protocol (Wang et al., 2017). Participants assumed a straight-arm push-up position with each hand placed on each force platform. The hands were positioned slightly wider than shoulder width, the wrists were maintained in a neutral position, the legs remained together, and the trunk and lower limbs were aligned throughout the test.

Following the start command, participants rapidly flexed the elbows to approximately 90° before performing a maximal explosive push to propel the upper body away from the force platforms. Upon landing, participants flexed the elbows to absorb the impact. During the flight phase, participants were instructed to maintain full elbow extension and straight body position to minimize compensatory movements.

Vertical ground-reaction forces recorded from the two force platforms were summed and analyzed using BioWare software. Peak force, peak power, and concentric-phase impulse were derived from the vertical force-time curve. Three trials were completed with 20 s of rest between trials, and the mean values were used for statistical analysis.

### Subjective fatigue

Subjective fatigue was assessed at P1 and P2 using a 100-mm visual analog scale (VAS) for fatigue (Lee et al., 1991). Participants indicated their current level of fatigue by placing a mark on a 100-mm horizontal line anchored by “no fatigue” (0 mm) and “extreme fatigue” (100 mm). The distance from the left anchor to the participant’s mark was measured and recorded as the fatigue score, with higher scores indicating greater perceived fatigue.

### Statistical analysis

All statistical analyses were performed using SPSS Statistics version 26.0. Data are presented as mean ± standard deviation (SD), and normality was assessed using the Shapiro–Wilk test. A 3 (condition) × 3 (time: P0, P1, P2) two-way repeated-measures ANOVA was used to analyze JPS, ROM, and upper-limb explosive performance, whereas fatigue VAS scores were analyzed using a 3 (condition) × 2 (time: P1, P2) repeated-measures ANOVA. Both condition and time were within-subject factors. When the assumption of sphericity was violated, the Greenhouse–Geisser correction was applied. Significant effects were followed by Bonferroni-adjusted pairwise comparisons. The reproducibility of the fatigue protocol across sessions was assessed using single-measure intraclass correlation coefficients (ICCs) from a two-way random-effects model with absolute agreement, with 95% confidence intervals. Effect sizes were expressed as partial eta squared (η_p_²). Statistical significance was set at *p* < 0.05.

## Results

All 15 participants completed the three experimental conditions, and all collected data were included in the final analysis. No adverse events related to the interventions were reported. Descriptive statistics for all outcome variables across conditions and time points are presented in **Supplementary Table S1**.

### Test–retest reliability of the exercise-induced fatigue protocol

The exercise-induced fatigue protocol demonstrated good-to-excellent reproducibility across the three experimental sessions. The intraclass correlation coefficient (ICC) was 0.915 (95% CI: 0.815–0.968) for total handstand push-up repetitions, 0.927 (95% CI: 0.839–0.972) for heart rate, and 0.758 (95% CI: 0.533–0.901) for rating of perceived exertion (RPE) (**Table 2**).

**Table 2:** Test–retest reliability of the exercise-induced fatigue protocol.

Variable	Single-measure ICC	95% CI	F(df1, df2)	P value
Handstand push-up total repetitions	0.915	0.815-0.968	33.604 (14, 28)	<0.001
Heart rate	0.927	0.839-0.972	38.345 (14, 28)	<0.001
RPE	0.758	0.533-0.901	11.247 (14, 28)	<0.001

ICC, intraclass correlation coefficient; CI, confidence interval; RPE, rating of perceived exertion. Single-measure ICCs were calculated using a two-way random-effects model with absolute agreement. P < 0.001 indicates that the ICC test reached statistical significance. Total repetitions refers to the cumulative number of handstand push-ups completed across all sets during each fatigue protocol.

### Fatigue VAS score

Fatigue VAS scores showed a significant condition × time interaction (F(2, 28) = 111.013, *p* < 0.001, η_p_² = 0.887), together with significant main effects of time (F(1, 14) = 378.893, *p* < 0.001, η_p_² = 0.964) and condition (F(2, 28) = 89.732, *p* < 0.001, η_p_² = 0.865). No between-condition differences were observed immediately after fatigue induction (P1; *p* > 0.05). Fatigue VAS scores decreased significantly from P1 to P2 in all three conditions (all *p* < 0.001). At P2, both PM interventions resulted in lower fatigue VAS scores than CTRL (both *p* < 0.001), with the 60 Hz condition producing lower scores than the 30 Hz condition (*p* = 0.038) (**Figure 3A**).

**Figure 3:**
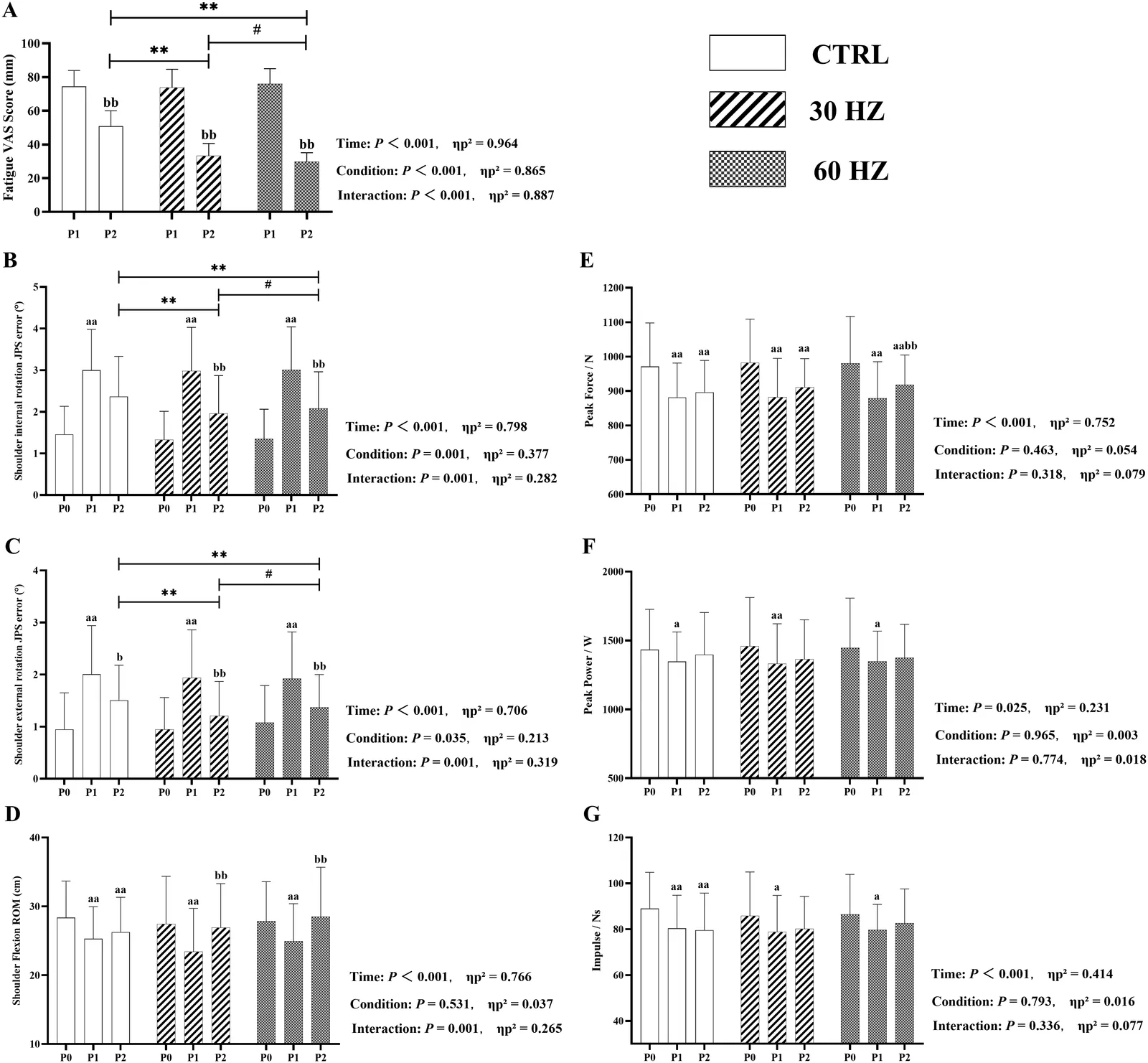
Changes in subjective fatigue, shoulder sensorimotor function, and upper-limb explosive performance across recovery conditions. **(A)** Subjective fatigue VAS score; **(B)** shoulder internal-rotation joint position sense (JPS) error; **(C)** shoulder external-rotation JPS error; **(D)** shoulder flexion range of motion (ROM); **(E)** peak force; **(F)** peak power; and **(G)** concentric-phase impulse. CTRL, seated-rest control; P0, baseline; P1, post-fatigue assessment; P2, post-recovery assessment. Subjective fatigue was assessed only at P1 and P2, whereas all other outcomes were assessed at P0, P1, and P2. Data are presented as mean ± SD. ^a^*p* < 0.05 and ^aa^*p* < 0.01 vs. P0; ^b^*p* < 0.05 and ^bb^*p* < 0.01 vs. P1; *p < 0.05 and **p < 0.01 vs. CTRL; ^#^*p* < 0.05 and ^##^*p* < 0.01 vs. 30 Hz.

### Shoulder internal rotation JPS error

Shoulder internal rotation JPS error demonstrated a significant condition × time interaction (F(4, 56) = 5.510, *p* = 0.001, η_p_² = 0.282), as well as significant main effects of time (F(2, 28) = 55.237, *p* < 0.001, η_p_² = 0.798) and condition (F(2, 28) = 8.482, *p* = 0.001, η_p_² = 0.377). No significant between-condition differences were found at baseline (P0) or immediately after fatigue induction (P1) (both *p* > 0.05). Internal rotation JPS error increased significantly from P0 to P1 and decreased from P1 to P2 in all three conditions (all *p* < 0.010). At P2, both PM interventions produced lower JPS errors than CTRL (both *p* < 0.001), while the 30 Hz intervention resulted in lower JPS error than the 60 Hz intervention (*p* = 0.010) (**Figure 3B**).

### Shoulder external rotation JPS error

Shoulder external rotation JPS error demonstrated a significant condition × time interaction (F(2.943, 41.207) = 6.555, *p* = 0.001, η_p_² = 0.319), together with significant main effects of time (F(2, 28) = 33.628, *p* < 0.001, η_p_² = 0.706) and condition (F(2, 28) = 3.781, *p* = 0.035, η_p_² = 0.213). No between-condition differences were observed at either baseline (P0) or immediately after fatigue induction (P1) (both *p* > 0.05). External rotation JPS error increased significantly from P0 to P1 in all three conditions (all *p* < 0.001) and decreased from P1 to P2 (all *p* ≤ 0.012). At P2, both PM interventions produced lower JPS errors than CTRL (*p* < 0.001 and *p* = 0.008 for 30 Hz and 60 Hz, respectively), with the 30 Hz intervention also resulting in lower JPS error than the 60 Hz intervention (*p* = 0.016) (**Figure 3C**).

### Shoulder flexion ROM

Shoulder flexion ROM showed a significant condition × time interaction (F(4, 56) = 5.061, *p* = 0.001, ηp² = 0.265) and a significant main effect of time (F(2, 28) = 45.781, *p* < 0.001, ηp² = 0.766), whereas the main effect of condition was not significant after Greenhouse–Geisser correction (F(1.410, 19.740) = 0.539, *p* = 0.531, ηp² = 0.037). No significant between-condition differences were observed at P0, P1, or P2 (all *p* > 0.05). Shoulder flexion ROM decreased significantly from P0 to P1 in all three conditions (all *p* < 0.001). From P1 to P2, ROM increased significantly following both the 30 Hz (*p* < 0.001) and 60 Hz (*p* = 0.001) conditions, whereas the change in the CTRL condition was not significant (*p* > 0.05) (**Figure 3D**).

### Upper-limb explosive performance

Peak force showed a significant main effect of time (F(2, 28) = 42.463, *p* < 0.001, η_p_² = 0.752), whereas neither the condition × time interaction (F(4, 56) = 1.207, *p* = 0.318, η_p_² = 0.079) nor the main effect of condition (F(2, 28) = 0.795, *p* = 0.463, η_p_² = 0.054) was significant. Peak force decreased significantly from P0 to P1 (*p* < 0.001) and increased from P1 to P2 (*p* = 0.019), although values at P2 remained lower than baseline (*p* < 0.001) (**Figure 3E**).

Peak power demonstrated a significant main effect of time (F(2, 28) = 4.209, *p* = 0.025, η_p_² = 0.231), whereas neither the condition × time interaction (F(1.975, 27.649) = 0.251, *p* = 0.774, η_p_² = 0.018) nor the main effect of condition (F(2, 28) = 0.036, *p* = 0.965, η_p_² = 0.003) reached statistical significance. Peak power showed a significant reduction from P0 to P1 (*p* = 0.046), whereas no significant differences were observed between P2 and either P0 or P1 (both *p* > 0.05) (**Figure 3F**).

Concentric-phase impulse showed a significant main effect of time (F(2, 28) = 9.881, *p* < 0.001, η_p_² = 0.414), whereas neither the condition × time interaction (F(4, 56) = 1.167, *p* = 0.336, η_p_² = 0.077) nor the main effect of condition (F(2, 28) = 0.233, *p* = 0.793, η_p_² = 0.016) was significant. Concentric-phase impulse was lower at both P1 (*p* = 0.010) and P2 (*p* = 0.012) than at baseline, with no significant difference between P1 and P2 (*p* > 0.05) (**Figure 3G**).

## Discussion

This randomized crossover trial examined the acute effects of 30 Hz and 60 Hz percussive massage (PM) on subjective fatigue, shoulder JPS, shoulder ROM, and upper-limb explosive performance following exercise-induced fatigue in high-level male artistic gymnasts. The findings partially supported our hypothesis. Compared with seated rest, both PM frequencies improved shoulder JPS recovery and reduced subjective fatigue, although the frequency associated with the greatest benefit differed between outcomes: 30 Hz produced greater improvements in JPS, whereas 60 Hz produced a greater reduction in perceived fatigue. Shoulder flexion ROM increased following both PM interventions, but the absence of between-condition differences precludes attributing this recovery specifically to PM. In addition, neither PM frequency provided an additional benefit for the recovery of upper-limb explosive performance. Collectively, these findings indicate that the acute effects of PM may be outcome- and frequency-specific rather than representing a uniform enhancement of post-fatigue recovery.

The standardized handstand push-up protocol produced a reproducible and sport-relevant upper-limb fatigue response. Good-to-excellent test–retest reliability was observed for the fatigue endpoint variables, while the changes from P0 to P1 were characterized by increased shoulder JPS error, together with reductions in shoulder ROM and upper-limb explosive performance. This combination of perceptual, sensorimotor, mobility, and performance changes indicates that the protocol imposed fatigue across multiple components of the upper-limb kinetic chain rather than affecting a single isolated outcome. Moreover, the absence of between-condition differences at P1 for most variables suggests that participants entered the intervention period in broadly comparable fatigue states across the three experimental conditions. This comparability strengthens the interpretation that subsequent differences in recovery were more likely related to the assigned recovery condition than to unequal fatigue induction.

Both PM frequencies produced greater reductions in fatigue VAS scores than seated rest, with the largest reduction occurring after the 60 Hz intervention. This finding indicates that PM may facilitate short-term perceived recovery following upper-limb fatigue in male artistic gymnasts. The present results differ from previous studies reporting limited benefits of PM over passive recovery, which may reflect differences in the mode of fatigue induction, the anatomical sites treated, and the sport-specific demands of the experimental task (Weerakkody et al., 2003; Alonso-Calvete et al., 2022). In the present study, fatigue was induced through repeated handstand push-ups, while PM was applied directly to the shoulder-girdle and upper-limb musculature involved in the fatiguing task. This close anatomical correspondence between the fatigued tissues and the treated regions may have increased the relevance of the mechanical and sensory stimulus to the athletes’ perceived recovery. The greater reduction in fatigue following 60 Hz PM may reflect stronger sensory stimulus that temporarily altered the perception of fatigue. Nevertheless, this interpretation remains tentative because neither perceptual processing nor neurophysiological responses to the two frequencies were directly measured.

Joint position sense provides an important sensorimotor basis for maintaining support stability, controlling body posture, and executing movement transitions in artistic gymnastics (Niespodziński et al., 2018). In the present study, exercise-induced fatigue increased both shoulder internal- and external-rotation JPS errors, indicating an acute deterioration in shoulder proprioceptive function. This finding is consistent with previous evidence that fatigue can impair proprioceptive acuity, potentially through alterations in peripheral sensory input and neuromuscular control (Proske, 2019). Following the recovery intervention, both PM conditions produced lower JPS errors than seated rest, suggesting that percussive stimulation may facilitate the short-term restoration of shoulder proprioceptive function after fatigue. Notably, JPS recovery was greater following the 30 Hz condition than the 60 Hz condition, indicating that a higher percussion frequency does not necessarily confer greater proprioceptive benefit.

One possible explanation is that different percussion frequencies generate distinct patterns of mechanical and sensory stimulation. PM may influence cutaneous, myofascial, and muscle-related afferent input through repeated mechanical stimulation of the treated tissues (Cheatham et al., 2021). Previous vibration research also suggests that the transmission and physiological effects of mechanical stimulation can vary according to stimulation frequency and the mechanical properties of the surrounding tissues (Wakeling et al., 2002; Yuan, 2022). Accordingly, the greater JPS recovery observed under the 30 Hz condition may reflect a more favorable pattern of peripheral sensory stimulation for the present task rather than a simple frequency-dependent increase in proprioceptive responsiveness. However, the underlying mechanisms cannot be determined from the present data because muscle spindle activity, afferent signaling, and central sensory processing were not directly assessed. Future studies incorporating electrophysiological or neurophysiological measures are needed to clarify the mechanisms underlying the frequency-dependent effects of PM on proprioceptive recovery.

Shoulder ROM is important for the execution of swinging and handstand skills in artistic gymnastics and may also be relevant to shoulder function and injury risk (Sleeper et al., 2016; Heck et al., 2021). In the present study, shoulder flexion ROM decreased following exercise-induced fatigue and subsequently increased from post-fatigue to post-recovery following both the 30 Hz and 60 Hz PM conditions. However, no significant between-condition differences were observed at any assessment time point. Therefore, although ROM improved during the recovery period following both PM conditions, the present findings do not demonstrate a PM-specific advantage over seated rest.

Previous studies have reported acute increases in joint ROM following percussive massage (Roberts et al., 2024). Mechanical percussion has been proposed to transiently influence soft-tissue stiffness, tissue compliance, and stretch tolerance, which could theoretically facilitate joint movement (Yang et al., 2023; Nakamura et al., 2024). Nevertheless, these mechanisms were not directly assessed in the present study, and the observed ROM changes may also reflect spontaneous recovery or repeated-testing effects. Accordingly, the current results should be interpreted as evidence of post-fatigue ROM recovery during the PM conditions rather than evidence that PM was superior to passive rest for restoring shoulder ROM.

Upper-limb explosive performance is important for pushing actions, take-off movements, and apparatus control in men’s artistic gymnastics. In the present study, neither PM frequency produced additional improvements in peak force, peak power, or concentric-phase impulse compared with seated rest, indicating that the recovery benefits observed for perceived fatigue and shoulder proprioception did not extend to upper-limb explosive performance. This finding differs from that report by Zhang (Zhang, 2017), who reported greater recovery of explosive performance following a 10-min, 30 Hz PM intervention. A possible explanation for this discrepancy is the difference in both fatigue induction and performance assessment. Previous work has often examined relatively localized fatigue and more isolated performance outcomes, whereas the present study induced fatigue using repeated handstand push-ups and assessed recovery with a ballistic push-up task. This task requires not only rapid force production but also coordinated multi-joint control, postural stability, and substantial neuromuscular involvement. Therefore, the acute effects of PM may not be sufficient to restore performance in such a complex movement within a short recovery period.

The dissociation between improvements in perceived fatigue and shoulder JPS and the absence of condition-specific changes in explosive performance further suggests that perceptual, sensorimotor, and mechanical aspects of recovery may not occur concurrently. Explosive performance is determined by multiple interacting factors, including neural activation, musculotendinous properties, intermuscular coordination, and the availability of high-energy phosphates (McMahon and Jenkins, 2002; Maffiuletti et al., 2016). Recovery from repeated high-intensity exercise has also been associated with phosphocreatine resynthesis, while different recovery strategies may influence subsequent power output to varying degrees depending on the metabolic and neuromuscular demands of the task (Connolly et al., 2003; Mendez-Villanueva et al., 2012). Accordingly, PM may facilitate local sensory or mechanical recovery without producing sufficient changes in the broader neuromuscular and metabolic processes required to enhance ballistic upper-limb performance. However, because neural activation, phosphocreatine resynthesis, and other physiological recovery processes were not directly measured in the present study, these explanations remain tentative.

Future studies should incorporate direct measures of neuromuscular and metabolic recovery to clarify why improvements in perceived or proprioceptive recovery do not necessarily translate into enhanced explosive performance. Comparisons between PM and other recovery strategies, including active recovery, may also help determine whether combined or multimodal approaches provide greater benefits for restoring repeated high-intensity performance (Connolly et al., 2003; Mendez-Villanueva et al., 2012). Nutritional strategies such as creatine supplementation may also be relevant to the broader recovery context because of their association with high-intensity exercise performance (Bird, 2003), although such approaches were beyond the scope of the present study.

### Limitations

The present study has several limitations that should be considered when interpreting the findings. First, only 15 high-level male artistic gymnasts were included. Although the randomized crossover design minimized the influence of inter-individual variability, the relatively small and homogeneous sample limits the generalizability of the findings to female gymnasts, youth athletes, and individuals with different levels of competitive experience. Second, the control condition consisted solely of seated rest, and no active recovery comparator, such as standardized manual massage, was included. Consequently, it remains unclear whether the observed benefits were attributable specifically to the mechanical vibration provided by PM or were partly influenced by non-specific recovery effects. Future studies should incorporate active comparison interventions to better distinguish the specific effects of PM from those of alternative recovery strategies. Finally, the proposed mechanisms underlying the frequency-specific effects of PM were inferred from changes in sensory input, soft tissue properties, and recovery physiology rather than being directly examined. Future investigations incorporating surface electromyography, electroencephalography, near-infrared spectroscopy, or muscle oxygenation measurements are warranted to further elucidate the neurophysiological and biomechanical mechanisms responsible for the observed recovery responses.

### Practical implications

From a practical perspective, the present findings suggest that a 10-min PM intervention may serve as a useful adjunctive recovery strategy during short recovery intervals in men’s artistic gymnastics, particularly when the primary objective is to reduce perceived fatigue or facilitate shoulder proprioceptive recovery. Importantly, the findings also indicate that the recovery benefits of PM are both outcome- and frequency-specific. Whereas 60 Hz appears to be more effective for reducing perceived fatigue, 30 Hz may be preferable for restoring shoulder joint position sense following fatigue. In contrast, neither frequency provided an acute advantage for restoring upper-limb explosive performance. Accordingly, practitioners may consider tailoring PM frequency to the specific recovery objective rather than expecting uniform benefits across all aspects of post-fatigue recovery.

## Conclusion

A 10-min PM intervention produced selective post-fatigue recovery benefits in high-level male artistic gymnasts. Compared with seated rest, both 30 Hz and 60 Hz PM reduced perceived fatigue and improved shoulder joint position sense, with 60 Hz producing a greater reduction in perceived fatigue and 30 Hz showing greater proprioceptive recovery. Shoulder ROM improved during recovery, but no PM-specific advantage over seated rest was demonstrated. Neither PM frequency provided additional benefits for upper-limb explosive performance. Collectively, these findings indicate that the acute recovery effects of PM are outcome- and frequency-dependent and support its use as a targeted adjunctive recovery strategy rather than a uniform means of enhancing post-fatigue function.

## Data Availability

The original contributions presented in the study are included in the article/**Supplementary Material**. Further inquiries can be directed to the corresponding author.
