# Supplementary material for: Acute effects of different percussive massage frequencies on shoulder proprioceptive recovery and upper-limb performance in male artistic gymnasts: a randomized crossover trial
Source: Front Physiol. 2026 Oct 28;17:1996859. doi: 10.3389/fphys.2026.1996859 (PMC13751405; doi:10.3389/fphys.2026.1996859)
Supplement: Supplementary file 1 [file Table1.doc]

**Supplementary Table S**1. Descriptive statistics for all outcome variables across experimental conditions and time points.

| Outcome variable | Condition | P0 | P1 | P2 |
| --- | --- | --- | --- | --- |
| Fatigue VAS score (mm) | CTRL | - | 74.53±9.40 | 50.86±9.19 |
|  | 30 Hz | - | 73.87±10.8 | 33.40±7.22 |
|  | 60 Hz | - | 76.00±9.04 | 29.87±5.28 |
| Shoulder internal rotation JPS error (°) | CTRL | 1.46±0.67 | 3.00±0.98 | 2.37±0.96 |
|  | 30 Hz | 1.33±0.68 | 2.98±1.05 | 1.96±0.91 |
|  | 60 Hz | 1.35±0.71 | 3.01±1.03 | 2.08±0.88 |
| Shoulder external rotation JPS error (°) | CTRL | 0.95±0.70 | 2.01±0.93 | 1.51±0.67 |
|  | 30 Hz | 0.95±0.61 | 1.94±0.92 | 1.21±0.66 |
|  | 60 Hz | 1.08±0.71 | 1.92±0.90 | 1.37±0.63 |
| Shoulder flexion ROM (cm) | CTRL | 28.37±5.31 | 25.30±4.65 | 26.27±5.06 |
|  | 30 Hz | 27.47±6.90 | 23.43±6.28 | 26.93±6.37 |
|  | 60Hz | 27.83±5.77 | 24.93±5.46 | 28.50±7.19 |
| Peak force (N) | CTRL | 971.18±126.74 | 881.43±100.03 | 896.49±92.30 |
|  | 30 Hz | 982.13±126.81 | 882.10±112.99 | 911.04±82.91 |
|  | 60 Hz | 980.47±136.52 | 878.92±106.14 | 918.52±86.32 |
| Peak power (W) | CTRL | 1433.00±293.16 | 1458.83±353.32 | 1446.33±360.93 |
|  | 30 Hz | 1347.47±214.87 | 1330.87±290.08 | 1347.67±220.02 |
|  | 60 Hz | 1397.47±305.60 | 1364.53±285.05 | 1375.20±242.42 |
| Concentric-phase impulse (N·s) | CTRL | 89.03±15.82 | 85.90±19.08 | 86.47±17.45 |
|  | 30 Hz | 80.40±14.43 | 78.83±15.97 | 79.77±11.05 |
|  | 60 Hz | 79.67±16.10 | 86.03±13.67 | 80.17±14.13 |

**Note:** Values are presented as mean ± SD. VAS = visual analogue scale; JPS = joint position sense; ROM = range of motion; P0 = baseline; P1 = after fatigue induction; P2 = after intervention/control. “—” indicates that fatigue VAS score was not assessed at P0.
